# Maize seed endophytes

**DOI:** 10.1111/mpp.13278

**Published:** 2022-11-22

**Authors:** Jason G. Wallace

**Affiliations:** ^1^ Department of Crop & Soil Science University of Georgia Athens Georgia USA

**Keywords:** host–microbe interactions, maize, maize–microbe interactions, microbiome, seed endophyte

## Abstract

Maize is a vital global crop, and each seed (kernel) hosts an ecosystem of microbes living inside it. However, we know very little about these endophytes and what their role is in plant production and physiology. In this Microreview, I summarize the major questions around maize seed endophytes, including what organisms are present, how they get there, whether and how they transmit across generations, and how they and the plant affect each other. Although several studies touch on each of these areas, ultimately there are far more questions than answers. Future priorities for research on maize seed endophytes should include understanding what adaptations allow microbes to be seed endophytes, how the host genetics and the environment affect these communities, and how maize seed endophytes ultimately contribute to the next generation of plants.

## Graphical abstract

Maize seed endophytes have complex interactions with their host, each other, and the environment. Understanding these interactions could lead to improved maize production.

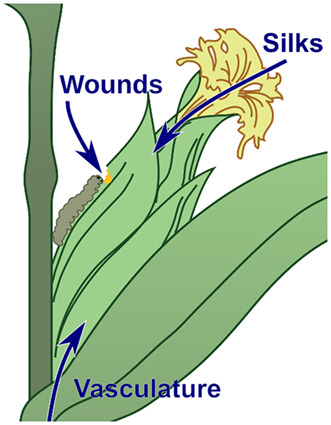

## 1 INTRODUCTION

The maize seed (kernel) is arguably the most important part of the plant. Although other tissues are clearly also needed, without seeds the plant is an agronomic failure and an evolutionary dead‐end. Assuming an average seed weight of 0.275 g (Clemens, n.d.), the 1.16 billion tonnes of maize harvested in 2020 (Food and Agriculture Organization of the United Nations, 2020) equates to roughly 4.2 *quadrillion* individual seeds—enough to trace Earth's orbit around the sun 900 times if laid end to end.

That's a lot of maize. Although much is known about seed composition (reviewed in Hamaker et al., 2019; Larkins, 2019; and Wang & White, 2019), we actually know very little about seeds as habitat. Every one of those seeds includes some number of microbes living inside, yet we know very little about what they are, why they are there, and how they impact the plant.

This Microreview collects what is known about maize seed endophytes: organisms living inside the seed but not causing obvious disease. It covers what seed endophytes are, how they get into the seed, what they do once there, and how they interact with each other and with their host. The ultimate goal is that by understanding this microcosm we can use it to make maize production safer, healthier, and more sustainable, even though there are large knowledge gaps we need to cross to get there. For a general overview of seed endophytes across different species, please refer to Shahzad et al. (2018).

## 2 WHAT ARE SEED ENDOPHYTES?

Seeds were originally thought to be sterile (Fernbach, 1888), but this view was discarded after many plant species were found to have microbes living in their seeds and related tissue (Mundt & Hinkle, 1976). Seed‐transmitted microbes are now recognized as a ubiquitous, if poorly understood, feature of plant reproduction. Some seed‐transmitted microbes even provide important functions for their host, such as nutrient solubilization in cacti (Puente et al., 2009), stress tolerance in cool‐season grasses (Clay & Schardl, 2002), and hormone and secondary metabolite production across many plants (reviewed in White et al., 2019).

The divisions between beneficial, commensal, and harmful microbes are fuzzy, and some microbes can shift between roles at different points (e.g., Degani et al., 2021; Kloepper et al., 2013; Rai & Agarkar, 2016; Zhou et al., 2018). For this Microreview, I use the broad definition that an endophyte is any organism that colonizes internal plant tissue for at least part of its lifetime (Hardoim et al., 2015). This thus includes beneficial and commensal organisms along with mild or latent pathogens; although this definition technically includes severe pathogens, this review will not focus on them because of the extensive literature already available (e.g., Degani, 2021; Goko et al., 2021; McGee, 1988). Organisms on the seed surface also fall outside this review simply because they are not endophytes.

## 3 WHAT MICROBES ARE IN MAIZE SEEDS?

**FIGURE 1:**
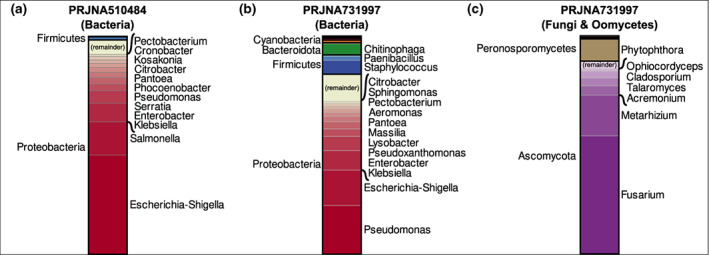
Distribution of seed microbes based on community sequencing. Community sequencing data was downloaded from the National Center for Biotechnology Information Sequence Read Archive accessions PRJNA510484 (Liu et al., 2020) and PRJNA731997(Johnston‐Monje et al., 2021), and taxonomically classified with Kraken2 (Wood et al., 2019) and Bracken (Lu et al., 2017). Each plot shows the relative fraction of the community based on bacterial 16S ribosomal RNA (a, b) or fungal internal transcribed spacer (c) amplicon sequencing; part (c) excludes bacterial reads (c.56%). Labels indicate taxa present at >1% of the total dataset, with phyla/divisions on the left and genera on the right

Many different microbes can grow in seed, and the maize seed community is distinct from other parts of the plant (Johnston‐Monje & Raizada, 2011). Only a handful of studies have looked at the maize seed endophyte community with modern deep sequencing methods (Johnston‐Monje et al., 2021; Liu et al., 2017, 2020; Majumdar et al., 2021; Santos et al., 2021), so most of our knowledge comes from isolation studies. However, based on both sequencing (Figure 1) and isolation (Tables 1 and 2), the bacteria of the maize seed are dominated by Proteobacteria, Actinobacteria, and Firmicutes, with the majority of fungi placed in the Ascomycota phylum. Archaea appear to be rare in seeds (e.g., only 0.04% of reads in Johnston‐Monje et al., 2021), and there appear to be no reports finding protists or nonpathogenic viruses in them.

**TABLE 1:** Bacterial endophytes isolated from maize seed

Phylum	Species	Citation
Actinobacteria	*Corynebacterium* spp.	Bodhankar et al. (2017)
Actinobacteria	*Frigoribacterium* spp.	Rijavec et al. (2007)
Actinobacteria	*Microbacterium* spp.	Johnston‐Monje and Raizada (2011); Rijavec et al. (2007)
Firmicutes	*Bacillus* spp.	Bodhankar et al. (2017); Bomfim et al. (2020); Johnston‐Monje and Raizada (2011); Pal et al. (2021); Rijavec et al. (2007)
Firmicutes	*Bacillus altitudinis*	Pal et al. (2021)
Firmicutes	*Bacillus amyloliquefaciens*	Gond et al. (2015)
Firmicutes	*Bacillus aquimaris*	Pal et al. (2021)
Firmicutes	*Bacillus cereus*	Pal et al. (2021)
Firmicutes	*Bacillus pumilus*	Pal et al. (2021)
Firmicutes	*Bacillus subtilis*	Degani et al. (2021); Gond et al. (2015); Pal et al. (2021)
Firmicutes	*Bacillus velezensis*	Pal et al. (2021)
Firmicutes	*Lysinibacillus* spp.	Pal et al. (2021)
Firmicutes	*Enterococcus* spp.	Johnston‐Monje and Raizada (2011)
Firmicutes	*Brevibacillus* spp.	Johnston‐Monje and Raizada (2011)
Firmicutes	*Paenibacillus* spp.	Bomfim et al. (2020); Johnston‐Monje and Raizada (2011); Rijavec et al. (2007)
Firmicutes	*Paenibacillus dendritiformis*	Pal et al. (2021)
Firmicutes	*Sphingomonas* spp.	Rijavec et al. (2007)
Firmicutes	*Staphylococcus* spp.	Bodhankar et al. (2017)
Firmicutes	*Staphylococcus arlettae*	Pal et al. (2021)
Bacteroidetes	*Sediminibacterium* spp.	Johnston‐Monje and Raizada (2011)
Proteobacteria	Unknown Betaproteobacteria	Rijavec et al. (2007)
Proteobacteria	*Burkholderia anthina*	Pal et al. (2021)
Proteobacteria	*Enterobacter* spp.	Johnston‐Monje and Raizada (2011)
Proteobacteria	*Pantoea* spp.	Johnston‐Monje and Raizada (2011); Rijavec et al. (2007)
Proteobacteria	*Methylobacterium* spp.	Johnston‐Monje and Raizada (2011)
Proteobacteria	*Acinetobacter* spp.	Bomfim et al. (2020)
Proteobacteria	*Pseudomonas* spp.	Pal et al. (2021)
Proteobacteria	*Pseudomonas aeruginosa*	Pal et al. (2021)

**TABLE 2:** Fungal endophytes isolated from maize seed

Division	Species	Reference
Ascomycota	*Chaetomium cochliodes*	Degani et al. (2021); Fisher et al. (1992)
Ascomycota	*Chaetomium subaffine*	Degani et al. (2021)
Ascomycota	*Cladosporium cladosporioides*	Fisher et al. (1992)
Ascomycota	*Epicoccum purpurascens*	Fisher et al. (1992)
Ascomycota	*Trichoderma asperellum*	Degani et al. (2021)
Ascomycota	*Fusarium oxysporum*	Fisher et al. (1992)
Ascomycota	*Fusarium proliferatum*	Degani et al. (2021)
Ascomycota	*Fusarium ventricosum*	Fisher et al. (1992)
Ascomycota	*Verticillium lecanii*	Fisher et al. (1992)
Ascomycota	*Alternaria alternata*	Degani et al. (2021)
Ascomycota	*Penicillium* spp.	Fisher et al. (1992)
Ascomycota	*Penicillium citrinum*	Degani et al. (2021)
Mucoromycota	*Rhizopus oryzae*	Degani et al. (2021)

Although Proteobacteria make up the majority of the community by sequencing, Firmicutes, particularly in the genus *Bacillus*, seem especially common among isolates (Bodhankar et al., 2017; Bomfim et al., 2020; Gond et al., 2015; Mundt & Hinkle, 1976; Pal et al., 2021; Rijavec et al., 2007; Yang et al., 2020). The reasons for this difference are unknown, but may have to do with many Firmicutes' ability to form spores for long‐term survival. *Pantoea* species are also common (Johnston‐Monje et al., 2014; Liu et al., 2013, 2017; Majumdar et al., 2021), and the only time course available of maize seed endophytes across development showed that both *Pantoea* and *Burkholderia* came to dominate the community as the seeds matured (Liu et al., 2013). This study also showed a decrease in seed endophyte diversity over time, which could be due to either actual loss of some endophytes or to a small number of them growing to dominate the community. Further experiments are needed to determine which of these is the case.

In addition to species, it would be useful to know the number of actual microbial cells living in seeds. Unfortunately, quantifying microbes in seeds is surprisingly hard. Isolation methods suffer from the same selectivity bias as they do elsewhere because only a subset of microbes will actually grow in the laboratory. Many isolation efforts get zero colonies from some seeds (Fisher et al., 1992; Marag & Suman, 2018; Mundt & Hinkle, 1976; Rijavec et al., 2007), although they often have better success after imbibing them for 24–48 h (Bacon & Hinton, 1997; Foley, 1962; Rijavec et al., 2007). This implies that imbibition either brings the microbes out of a dormant state or results in a large increase in their numbers, or (most likely) some combination of the two. (Readers interested in isolation methods are referred to the extensive review of Chowdhury et al., 2019.) So, the exact number of microbes in a seed is still unknown, although we can probably assume it is small.

Another issue with seed endophytes is that there are very few genome sequences available. The only exceptions appear to be a trio of *Pantoea ananatis* isolates that have different effects on the plant despite highly similar genomes (Sheibani‐Tezerji et al., 2015), and the beneficial endophyte *Bacillus mojavensis* RCC101 (Gold et al., 2014). Until more genomes are available from both bacterial and fungal endophytes, we can only assume that maize seed endophytes probably share genomic features with other plant‐associated microbes. This would include enrichment for carbohydrate metabolism (Levy et al., 2017), which covers both metabolism of free sugars and also genes involved in infecting the plant (cellulases, pectin lyases, expansins, etc.). Genes associated with beneficial effects on the plants (siderophores, plant hormone production, phosphate solubilization, etc.) or inhibiting pathogens (chitinase, antibiotics, etc.) may also be present, but probably vary considerably from one microbe to another. For example, the beneficial endophyte *B. mojavensis* RCC101 contains genes for surfactin and fengycin (two antifungal products), quercetin dioxygenase (degrades plant antimicrobial exudates), and acetoin reductase (produces a plant growth‐promoting volatile compound) (Gold et al., 2014). Ultimately, having a database of genomes from seed endophytes would let us better understand what they share with other plant‐associated microbes and what, if anything, makes them unique.

## 4 WHERE ARE SEED ENDOPHYTES?

Endophytes have been localized to all the interior regions of the seed, including the pedicel, abscission layer, endosperm, radicle, and embryo (Fisher et al., 1992; Mitter et al., 2017).

Because seed‐inherited microbes are difficult to track, little is known about where they end up in the mature plant. Transmission across multiple generations (see below) implies they must at least be in the stalk and aboveground vasculature. Electron microscopy of sterile, germinating seeds shows microbes in the pericarp, endosperm, radicle, and germinating root surface (Santos et al., 2021), and the beneficial microbe *B. mojavensis* RCC101 was seen to cover the root epidermis after germination (Bacon et al., 2001). The presence of endophytes on the root surface implies that microbes can move from the seed out into the rhizoplane and rhizosphere, a conclusion supported by other work (Johnston‐Monje & Raizada, 2011; White et al., 2019). This indicates that seed endophytes can spread both throughout the plant and into the local environment, two different routes for the endophyte to propagate itself.

## 5 HOW DO ENDOPHYTES GET INTO THE SEED?

**FIGURE 2:**
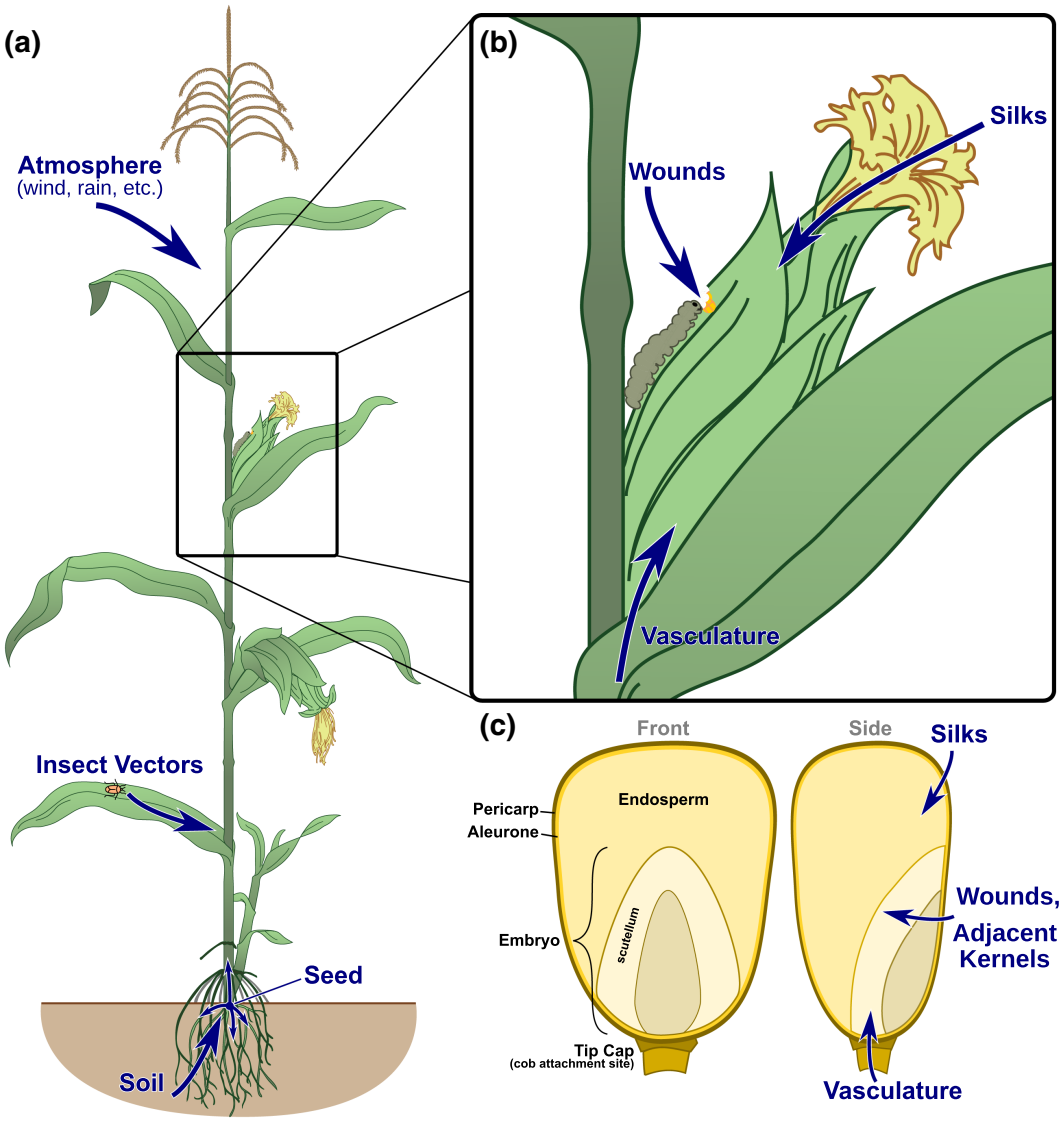
Routes of entry for seed endophytes. (a) Maize endophytes can enter the plant through several routes, including being inherited via seed, invading from the soil and air, or being transmitted by insects or other vectors. (b) These endophytes can then potentially transmit to the seed via the vasculature, or seeds can be directly infected via the silks or wounds. (c) Diagram of a maize seed, showing the major tissue types and routes of invasion from (b)

Endophytes have several potential routes to get into seeds (Figure 2) (reviewed in Rodríguez et al., 2018; Shade et al., 2017). The main routes are transmission from the parent plant via vasculature or acquisition from the environment via silks or wounds. A key distinction here is whether the endophytes are vertically or horizontally transmitted. Vertically transmitted microbes come from one of the seed's parents. Most of these microbes probably invade the seed through the vasculature, although evidence in other plants (reviewed in Frank et al., 2017) indicates that microbes can also associate with pollen and thus potentially come from the male parent as well. Whether this actually occurs in maize has not been shown, although there are some hints that it may (e.g., Wu et al., 2022).

Horizontal transmission is probably most common through the silks, as these represent a direct route from the environment to the developing seed. It has been suggested that the silk microbiome could be selected to control this route (Khalaf et al., 2021) because it is a favoured entry for fungal pathogens. Silks have even been used to artificially introduce endophytes into seeds (Mitter et al., 2017), and the ease and high success rate (90% in wheat, not reported in maize) implies it is probably common in nature. Horizontal transmission could also occur via wounds (such as from insects), although the author could find no studies on this for nonpathogenic endophytes.

## 6 HOW WELL DO SEED ENDOPHYTES TRANSMIT ACROSS GENERATIONS?

Traditionally, it was thought that seeds represented a minor source of microbes for the new plant, with most microbes (endophytes and otherwise) coming from the soil (McInroy & Kloepper, 1995). These results were based on isolating live microbes from seeds and plants. More recent work with high‐throughput sequencing challenges this view, however, with evidence that a large fraction of a plant's microbiota can be inherited via seed (Johnston‐Monje et al., 2016, 2021).

Because seed endophytes are arguably the best way maize could pass a microbiome on to progeny, the natural question is, how well does it actually do so? Many studies find relatively poor transmission of endophytes through the generations. For example, Johnston‐Monje and Raizada (2011) found that only nine of 26 genera were successfully transmitted, while a later study by the same group found transmission fidelity of 50%–75% (Johnston‐Monje et al., 2014). A test with β‐glucuronidase (GUS)‐labelled *Fusarium* found only 35% of seeds infected with the labelled strain (Bacon et al., 2001). These results match work in rice, where only 45% of microbes from the parent generation were also found in the progeny (Hardoim et al., 2012). In some cases, this reduction could be due to loss of endophytes during storage, as endophytes are known to lose viability faster than the seed itself (Bacon & Hinton, 2019; Mitter et al., 2017; Mundt & Hinkle, 1976).

Despite this, some endophytes do seem capable of faithful transmission. For example, GUS‐labelled *Fusarium* showed faithful transmission through three generations of maize (Bacon & Hinton, 2019). Similarly, when GUS‐labelled *Paraburkholderia phytofirmans* PsJN was introduced via silks, labelled bacteria were found in both infected seeds and the subsequent plant (Mitter et al., 2017). Ideally, we would also want to see transmission from that plant into its own seeds, but this was not tested.

Another reason for low seed transmission is that getting into seeds could be a stochastic process, and a given endophyte may only make it into a subset of seeds. Unfortunately, no analysis of the general seed microbiome and how it varies within a maize ear is available. A recent study on common bean (*Phaseolus vulgaris*) found relatively consistent transmission within a plant, with plant‐to‐plant variability being much higher than seed‐to‐seed within the same plant (Bintarti et al., 2022). This implies that maize seeds may be fairly consistent within a plant, although actual studies are needed to confirm this.

Different studies have come to conflicting conclusions as to how important the maize seed microbiome is for the next generation. Early studies found that surface‐sterilized seeds grown on water agar had two to four orders of magnitude fewer microbes than the same seeds grown in soil, implying that soil was the major source of plant microbes (McInroy & Kloepper, 1995). More recent studies with sequencing‐based approaches found that seeds had a larger effect on plant microbiomes than soil type, at least in the first 20 days (Johnston‐Monje et al., 2014). Later these authors speculate that the importance of seed transmission could drop the longer the plant lives and can be colonized from the environment (Johnston‐Monje et al., 2016). A recent follow‐up study involved maize and 17 other plant species growing in both sterile sand and nonsterile soil (Johnston‐Monje et al., 2021). It found that most microbes were shared between these conditions, implying they came via seeds. Because these seeds were not surface‐sterilized, it is unknown how many of the microbes were endophytes versus living on the seed surface. Combining these results with the low transmission rate of many endophytes (see above) creates a paradox: we cannot predict which microbes make it into a seed, but those that do appear to have a significant impact on the seedling microbiome. Resolving this apparent contradiction will require specific research on both how endophytes get into the seed and how they affect the resulting plant.

## 7 HOW WELL CAN WE MANIPULATE MAIZE SEED ENDOPHYTES?

A key goal for using endophytes in agriculture is that we want some way to manipulate them. Generally, the seed endophyte community is manipulated by removing existing endophytes, adding new ones, and/or modifying the endophytes themselves.

Removing existing endophytes is deceptively tricky. Surface‐sterilization—usually via bleach and ethanol—can remove microbes on the exterior, but disinfecting the seed interior while keeping it alive is hard. The main methods are hot‐water baths (Bacon et al., 1994; Daniels, 1983) or antibiotic treatments (Pal et al., 2021). To our knowledge, no one has shown that either of these procedures fully removes the endophyte population, only that they significantly reduce it. Although various groups describe work with axenic (germ‐free) maize (e.g., Groleau‐Renaud et al., 1998; Hussain et al., 2013; Niu et al., 2017; Shaharoona et al., 2006), this usually refers to surface‐sterilization and sterile growing conditions; the presence of seed‐transmitted endophytes is rarely, if ever, checked for.

Putting endophytes *into* seeds, on the other hand, is rarely attempted. Although there are many examples in the literature of people inoculating maize seeds with microbes (e.g., Bano et al., 2013; Casanovas et al., 2002; Oliveira et al., 2017; Viruel et al., 2014), the vast majority of these are not actually trying to get microbes inside the seed itself. Instead, the goal is to put inoculum (live culture, spores, etc.) on the seed surface so that the microbes are positioned to infect the new plant during germination (O'Callaghan, 2016). In theory these microbes could eventually end up in seeds, although this is rarely tested. More direct infection of seeds can be done by inoculating silks, as has been done with *Paraburkholderia phytofirmans* PsJN (Mitter et al., 2017), although this needs to be tested with additional species.

Finally, the endophytes themselves can be manipulated. This usually takes the form of tagging them with a label, such as green fluorescent protein (GFP) (Johnston‐Monje & Raizada, 2011; Mousa et al., 2015) or GUS (Bacon et al., 2001; Bacon & Hinton, 2019; Mitter et al., 2017). Unfortunately, not all endophytes can be transformed; for example, Mousa et al. (2015) only succeeded in putting GFPuv in one of four endophytes they worked on. When transformation fails, isolating natural rifampicin mutants (e.g., Bodhankar et al., 2017) may work instead, at least for bacteria, because they can be identified by plating onto selective media. In all of these cases, the goal of manipulation is to track the microbes in planta; the author is not aware of any cases where seed endophytes have been altered to test gene function or plant/seed associations. Such experiments will eventually be necessary to understand how seed endophytes work with and within the plant.

## 8 HOW DO SEED ENDOPHYTES AFFECT THE MAIZE PLANT?

Endophytes in general are often studied for their growth‐promoting capacities, and seed endophytes are no exception. Seed‐transmitted endophytes can affect the plant either directly or indirectly through their interactions with other organisms.

Direct interactions include endophyte functions that are traditionally associated with plant‐growth‐promoting activity, such as phosphate solubilization, nitrogen fixation, siderophore production, and plant hormone synthesis (Gold et al., 2014; Gond et al., 2015; Pal et al., 2021; Ravichandran et al., 2021; Sandhya et al., 2017; Siddique et al., 2022). The presence of these genes or their activity in vitro does not, unfortunately, guarantee they actually increase plant growth in vivo. For example, one survey of seed endophytes found that most of the isolates with supposed growth‐promoting activities actually *decreased* the growth of plants (Johnston‐Monje & Raizada, 2011). A similar result was found by Bomfim et al. (2020), where 11 out of 51 seed isolates actually reduced germination and root growth. Sometimes the relationship is more complex, such as how a symptomless *Fusarium* infection was shown to reduce seedling growth after 7 days, but by 28 days the infected plants had recovered or even surpassed uninfected ones (Yates et al., 1997).

When seed endophytes are found to promote growth, it is usually by increasing the size of roots, shoots, leaves, and so on (Bacon et al., 2001; Bomfim et al., 2020; Degani et al., 2021; Siddique et al., 2022). Other identified benefits include better germination (Bacon et al., 2001; Bomfim et al., 2020; Degani et al., 2021), drought tolerance (Siddique et al., 2022), and ion accumulation (Bomfim et al., 2020). Seed endophytes can also induce defence genes, such as a *Bacillus subtilis* isolate that induced the genes *PR‐1* (antifungal protein) and *PR‐4* (chitinase), and slightly increased *Sod‐2* (superoxide dismutase) (Gond et al., 2015).

In contrast to these direct interactions between seed endophytes and the plant, many of their impacts occur indirectly through their interactions with other microbes. Much of the focus in this area is directed toward controlling pathogens (Chulze et al., 2015; Degani et al., 2021; Liu et al., 2016; Rijavec et al., 2007; Wicklow et al., 2005). Many of these interactions are tested in vitro, however, so it is usually not known if they actually impact the disease in realistic conditions. An exception found that endophytes antagonistic to *Fusarium* and other fungal pathogens did result in a significant yield increase when disease pressure was high, and that they also reduced mycotoxins during storage through unknown mechanisms (Mousa et al., 2015). Interestingly, the most consistent of these endophytes all came from wild teosintes, potentially supporting the hypothesis that domestication and breeding reduced maize's dependence on beneficial microbes (Berg & Raaijmakers, 2018; Pérez‐Jaramillo et al., 2016; Soldan et al., 2021). Another study isolated 11 endophytes for their ability to antagonize late wilt (*Magnaporthiopsis maydis*) in vitro, two of which also improved performance in greenhouse trials (Degani et al., 2021).

The mechanisms of how seed endophytes interact with other microbes varies. *Fusarium*, for example, produces fusaric acid, which among other things interferes with quorum sensing in bacterial competitors (Bacon & Hinton, 2019). In the other direction, several *Bacillus* species inhibit *Fusarium* by producing lipopeptides (Gond et al., 2015; Yang et al., 2020), while *B. mojavensis* RCC101 does the same by producing surfactin and fengycin (Gold et al., 2014; Hinton & Bacon, 1995). Pal et al. (2021) found a *Bacillus velezensis* isolate (ZMW8) with multiple known antifungal genes (bacillomycin and Iturin A) that was able to reduce *Fusarium* infection of seeds by 90%. The authors also found that seeds treated with bacteria‐specific antibiotics showed fungal growth during germination, while untreated ones did not, implying at least some bacteria in the seeds helped keep fungal pathogens under control (Pal et al., 2021). This antagonism may also explain why bacteria and fungi have different distributions in grown plants, so that there are more bacteria closer to the soil and more fungi further from it (Fisher et al., 1992; Hallmann et al., 1997), although to our knowledge this remains speculative.

## 9 HOW DOES MAIZE GENETICS AFFECT SEED ENDOPHYTES?

The degree to which maize genetics affects seed endophytes—or any endophytes, for that matter—is still an open question. Most studies on plant genetic effects end up confounding plant genotype with other factors like seed sources or fungicide treatments (e.g., Johnston‐Monje et al., 2014, 2016; Majumdar et al., 2021). Other studies suffer from low sample size, making firm conclusions difficult (e.g., Liu et al., 2017, 2020). Despite these shortcomings, we can draw some conclusions.

First, there is a small amount of evidence that the pollen parent can affect microbes in the seed (Liu et al., 2017). This could occur through typical xenia effects (where the genotype of the seed changes its phenotype) or from microbes hitchhiking with the pollen, although in either case confirmation is needed from additional studies.

In addition, several studies have found differences in seed endophytes between domestic maize and its ancestor teosinte, as shown generally by Johnston‐Monje and Raizada (2011). Desjardins et al. (2000) found a much lower rate of infection of symptomless *Fusarium verticillioides* infection in *Zea parviglumis* (4%) relative to modern maize (100%), although at least some of that could be due to the physical differences between them (e.g., presence of a protective fruitcase) (Bacon & Hinton, 2019). There is some speculation that beneficial endophytes could have been lost over the course of domestication (Berg & Raaijmakers, 2018; Pérez‐Jaramillo et al., 2016; Soldan et al., 2021). Although the author is unaware of any systematic tests of this in maize, Mousa et al. (2015) did find that their three best biocontrol endophytes against *Fusarium graminearum* all came from teosintes. Taken together, these results imply that domestication altered at least some aspects of how maize interacts with seed endophytes, although the mechanisms and consequences are still mostly unknown.

## 10 CONCLUSIONS AND FUTURE DIRECTIONS

From the above information, we can conclude that maize endophytes are probably ubiquitous, and that we still know very little about them and their roles in the plant. There are far more questions than answers in this area, with major knowledge gaps in every area.

Although there are many areas open for research about maize seed endophytes, there are three questions that seem most important in the short term:

Question 1: What are the common traits among maize seed endophytes? The seed is a specialized environment and surviving there long enough to reach germination requires special adaptations. How do microbes get in the seed in the first place, what adaptations allow them to survive there, and how do they protect their niche from competitors?

Question 2: How do environment and host genetic factors affect the seed endophyte community? Seed endophytes do not exist in a vacuum, and both the environment and the host presumably have significant impacts on the microbial community. What role does each of these play and how do they interact? Given that a single endophyte can sometimes transition among beneficial, commensal, and harmful lifestyles, it would be especially important to determine what controls that switching, and if we could manipulate that to our benefit.

Question 3: What is the effect of seed endophytes on the next generation of plants? This is the most practical question, as it lets us manipulate plant performance via seed endophytes. Growth promotion, stress tolerance, and disease resistance are the goals, but we also need to be testing for complex interactions with other microbes, nutrient utilization, seed viability, and the like. Ultimately, this is the question of how seed endophytes affect maize, and how we can use that to our advantage.

Answering these questions will take a significant amount of work, and will probably spawn yet more questions about these communities. Yet given the importance of maize to global agriculture and the sheer amount of seeds (and thus seed endophytes) that we produce every year, answering these questions could open new avenues to more reliable and sustainable maize production.

## Data Availability

Data sharing is not applicable to this article as no new data were created or analysed.
